# Sanguinarine ameliorates rheumatoid arthritis by activating the Hippo-YAP/TAZ pathway to induce apoptosis and suppress inflammation in fibroblast-like synoviocytes

**DOI:** 10.3389/fphar.2026.1887163

**Published:** 2026-09-28

**Authors:** Jun Cai, Yong-Gu Cai, Jing-Yi Luo, Mei-Jun Xiao, Shuai Li, Qiao-Yuan Ye

**Affiliations:** 1 The Second Clinical Medical College, Guangdong Medical University, Dongguan, China; 2 Innovation Center of Cardiometabolic Diseases, Guangdong Medical University, Dongguan, China

**Keywords:** apoptosis, HIPPO-YAP/TAZ, rheumatoid arthritis, ROS, sanguinarine

## Abstract

**Introduction:**

Rheumatoid arthritis (RA) is a chronic autoimmune disease with progressive joint damage, functional disability and systemic comorbidity. Available therapies are not satisfactory, including low rates of remission and about 30% of patients with poor response to first-line treatment. Thus, more effective therapeutic strategies are urgently needed. Sanguinarine, a benzophenanthridine alkaloid primarily isolated from Sanguinaria canadensis and other members of the Papaveraceae family, has been documented to possess notable anti-bacterial, anti-inflammatory, and anti-neoplastic properties. Nevertheless, its potential utility as a targeted therapeutic modality for RA has yet to be systematically investigated.

**Methods:**

Here, we demonstrate that sanguinarine can effectively ameliorate the development of adjuvant-induced arthritis in rats and decrease arthritis scores, attenuate the inflammatory response, and prevent bone and joint damage.

**Results:**

Flow cytometry and ELISA both confirmed its anti-inflammatory activity, reducing the levels of PGE2, TNF-α, IL-1, and IL-6. In human fibroblast-like synoviocytes from RA patients (HFLS-RA), sanguinarine triggered cell apoptosis accompanied by elevated intracellular ROS, loss of mitochondrial membrane potential and activation of caspase-3, and activated the Hippo/YAP pathway both *in vitro* and *in vivo*.

**Discussion:**

These results suggest that sanguinarine ameliorates RA by activation of the Hippo-YAP/TAZ pathway to induce apoptosis and suppress inflammation, highlighting its potential as a novel therapeutic candidate for RA.

## Graphical Abstract

**Figure:**
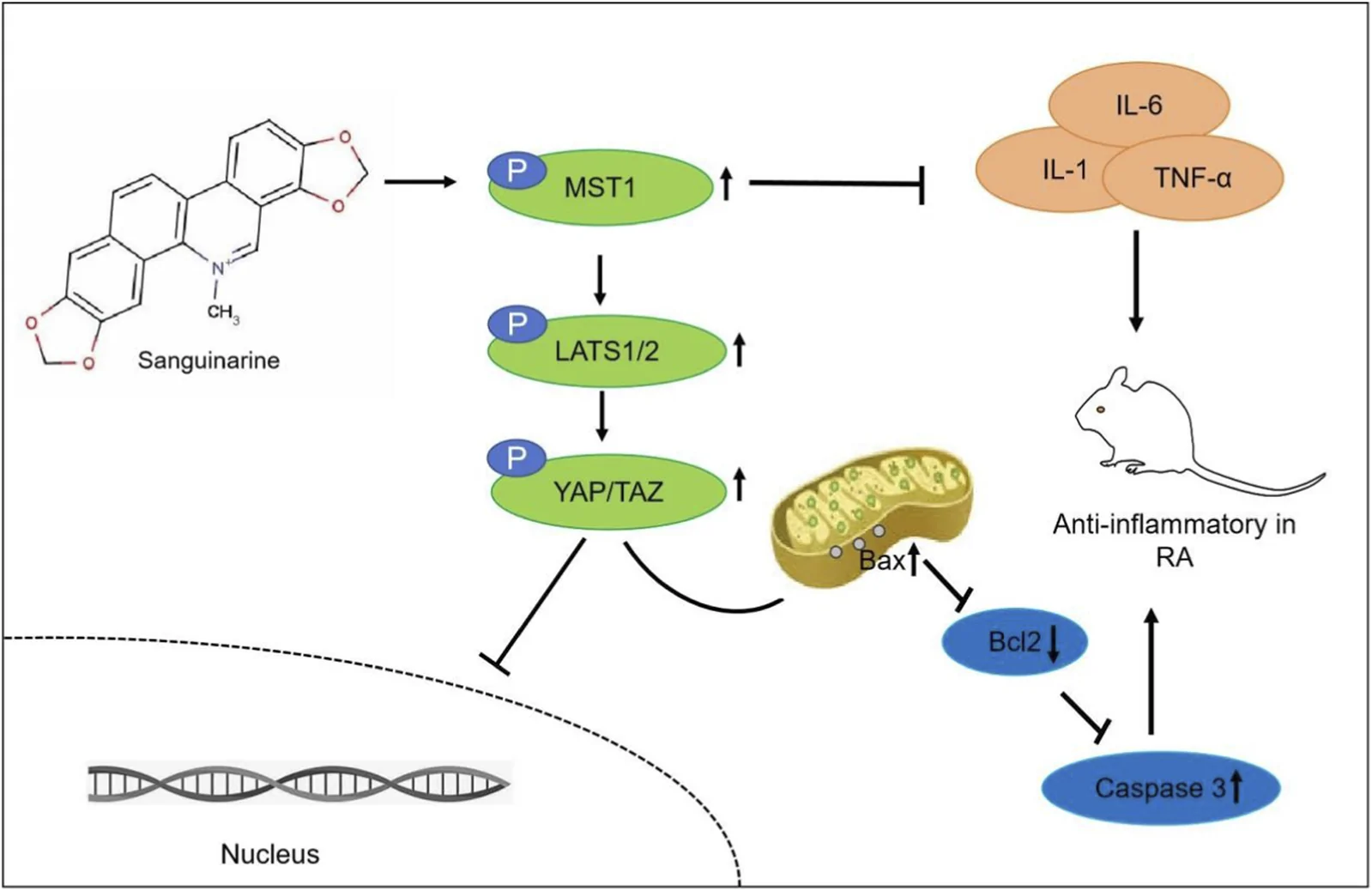
Sanguinarine ameliorates rheumatoid arthritis by activating the Hippo-YAP/TAZ pathway to induce apoptosis and suppress inflammation.

## Introduction

Characterized by persistent inflammatory synovitis, rheumatoid arthritis (RA) is a chronic, systemic autoimmune disorder (Di Matteo et al., 2023). RA brings a huge burden of disease, characterized by progressive physical disability, increased mortality rates, and significant socio-economic costs (Aletaha and Smolen, 2018; De Stefano et al., 2023). RA can occur in any age group, but its incidence rate increases significantly after 40 years of age. Its etiology has not been fully elucidated. It should be noted that there is currently no cure for RA (Burmester and Pope, 2017; Smith and Berman, 2022). At present, the treatment strategy for this disease has undergone substantial changes, shifting from traditional nonsteroidal anti-inflammatory drugs (NSAIDs), glucocorticoids (GC), and traditional disease-modifying antirheumatic drugs (DMARDs) to biologics targeting specific inflammatory cytokines and pathways, such as tumor necrosis factor alpha (TNF-α), cyclooxygenase (COX), and interleukin (IL) signaling (Prasad et al., 2023; Sahin et al., 2025). For instance, the IL-6 receptor blocker sarilumab, approved by the Food and Drug Administration (FDA) in 2017, has shown clinical efficacy in the treatment of RA (Aly and Furst, 2017). Although these targeted drugs can effectively alleviate pain, inhibit inflammation, and slow down disease progression, there are also significant limitations. For example, high medical costs, a series of immune-related adverse events, and serious side effects such as cardiovascular complications, gastrointestinal ulcers, and infections associated with long-term immune suppression (Huang et al., 2021; Wei and Chu, 2022). Therefore, there is an urgent need to find safer and more affordable therapeutic drugs.

Traditional Chinese medicine (TCM) has a history of thousands of years in treating various joint diseases in Asia. Sanguinarine, a bioactive alkaloid derived from traditional Chinese medicinal plants including *Chelidonium majus*, *Corydalis edulis Maxim, Eomecon chionantha,* and *Macleya cordata*, exhibits broad-spectrum pharmacological properties encompassing anti-microbial, anti-inflammatory, anti-oxidant, anti-tumor, and pro-apoptotic activities (Fu et al., 2018; Lu et al., 2022). In addition, sanguinarine has been shown to promote apoptosis in various types of cells. For instance, it inhibits the progression of oral squamous cell carcinoma via suppression of the PKM2/TFEB axis, disruption of lysosomal function, and subsequent autophagic blockade. In hepatocellular carcinoma, sanguinarine exhibits anti-tumor activity by regulating energy metabolism through the PKM2/β-catenin signaling pathway (Kong et al., 2024; Peng et al., 2025). However, whether sanguinarine exerts effects in rheumatoid arthritis fibroblast-like synoviocytes (RA-FLSs) and the underlying mechanisms are still unclear.

The Hippo-YAP signaling pathway primarily functions to regulate embryonic development, cell apoptosis, cell differentiation and organ size control (Fu et al., 2022). In addition, the functions of the Hippo-YAP axis in RA have been understudied. One prior study reported that YAP/TAZ were transcriptionally active in arthritis (Caire et al., 2021). Moreover, the study indicated that abnormal activation of the Hippo-YAP/TAZ cascade disrupts the homeostasis of articular cartilage, which further contributes to cartilage injury (Deng et al., 2018). Nevertheless, the precise role of the Hippo-YAP cascade in arthritic pathogenesis, as well as whether sanguinarine exerts its effects via this signaling axis, remains largely elusive.

In this study, we elucidated that sanguinarine induces apoptosis and suppresses inflammation in Adjuvant-Induced Arthritis (AIA) rats and HFLS-RA cells via Hippo-YAP/TAZ pathway activation. This indicates a mechanistic link between sanguinarine-triggered Hippo-YAP/TAZ signaling activation and HFLS cell death in RA. Furthermore, our findings highlight the therapeutic potential of sanguinarine in treating RA.

## Materials and methods

### Materials

The HFLS-RA cell line was purchased from the ATCC(United States). Sanguinarine was purchased from TargetMol (Cat. No.: T2781), penicillin/streptomycin (Cat. No.: P4333), N-acetyl-L-cysteine (NAC, Cat. No.: A9165) and DMSO (Cat. No.: D8418) were obtained from Sigma-Aldrich (St. Louis, MO, United States). MTT (Cat. No.: M6494), FBS (Cat. No.: A5256701), BSA (Cat. No.: 30066575), Dulbecco’s Modified Eagle’s Medium (DMEM, Cat. No.: 12430054), and Trypsin (0.25%, Cat. No.: 15050065) were obtained from Gibco (Grand Island, United States). Enzyme-linked immunosorbent assay (ELISA) kits for prostaglandin E2 (PGE2, Cat. No.: CSB-E07967r) was provided by SUSA Bio (Wuhan, China), tumor necrosis factor-alpha (TNF-α, Cat. No.: ER006-96), interleukin-1 (IL-1, Cat. No.: ER008-96), and interleukin-6 (IL-6, Cat. No.: ER003-96) were provided by ExCell Bio (Shanghai, China). Reagents, including JC-1(Cat. No.: C2006), TUNEL (Cat. No.: C1088), Caspase 3 Activity Assay Kit (Cat. No.: C1116), LDH cytotoxicity kit (Cat. No.: C0017), and Annexin V-FITC/PI apoptosis detection kit (Cat. No.: C1062) were purchased from Beyotime Biotechnology (Shanghai, China). Primary antibodies BCL-2, BAX, and GAPDH were sourced from Santa Cruz Biotechnology (Dallas, United States). Antibodies against p-MST1/2, p-LATS1/2, p-YAP and p-TAZ were purchased from Cell Signaling Technology. The reactive oxygen species (ROS) probe, DCFH-DA, was obtained from Invitrogen (Carlsbad, United States). HRP-conjugated anti-rabbit IgG secondary antibody was purchased from Promega (Madison, United States). MST1-targeted siRNA was obtained from Ribobio Co., Ltd. (Guangzhou, China). XMU-MP-1 was purchased from Proteintech (Wuhan, China, Cat. No.:CM05215).

### Establishment of the AIA rat model

Adult Sprague-Dawley (SD) rats were purchased from Charles River and housed in a dedicated germ-free room at 24 °C–26 °C, 12 h/dark light cycle with water and food supplied *ad libitum*. As described previously, rats (120–150 g) via a single intradermal injection at the tail base with 0.2 mg heat-killed *Mycobacterium tuberculosis* emulsified in 100 μL Complete Freund’s Adjuvant (CFA) (Cai et al., 2022). Animals were randomized into six experimental groups: (1) untreated, non-arthritic controls (Normal); (2) AIA model controls (CFA + daily PBS); (3) positive controls (CFA + methotrexate, 7.5 mg/kg/week, oral gavage); and (4–6) sanguinarine-treated groups (CFA +2.5, 5, or 10 mg/kg sanguinarine daily via i.p. injection). All treatments commenced on day 1 post-immunization and continued for 30 consecutive days. Arthritis severity was assessed every 3 days by macroscopic scoring of all four paws and the tail (0–4 per site; maximum total score 20). Hind paw volume was quantified by water displacement plethysmometry (Ugo Basile 37,140). On the 30th day, euthanize each group of animals, collect the spleen, thymus, and liver of each group of mice, weigh them, and calculate the organ index (organ wet weight (mg)/body weight (g) × 100%). Perform high-resolution micro-CT imaging (Bruker Skyscan) on the hind legs of rats. The collected images are reconstructed into a 3D model for detailed bone structure analysis. All animal procedures have been approved by the Ethics Committee of the Macau University of Science and Technology (approval number AL003/DICV/DIS/2021) and strictly follow the established guidelines for the care and use of experimental animals.

### Plasma cytokine measurement

Blood was collected from the orbit and centrifuged at 3,000 rpm for 15 min at 4 °C to obtain plasma. The levels of PGE2, TNF-α, IL-1β, and IL-6 were quantified according to the manufacturer’s experimental protocol. Measure the absorbance at 450 nm. The experiment was repeated at least three times.

### HE staining and safranin O staining

After decalcification of the hind legs of SD rats, the leg tissues of rats in each group were paraffin-embedded tissue sections, and the tissues were dewaxed and dehydrated with different concentrations of xylene, ethanol, and distilled water. Subsequently, paraffin tissue sections were stained with hematoxylin, and then briefly soaked in distilled water and 70% ethanol containing 1% HCl (3 s) for differentiation. The slices were washed in tap water for 30–60 min, then re-stained with eosin for 8 s, and then dehydrated with graded ethanol. Finally, the slices were removed in xylene I (2 min) and xylene II (2 min) and fixed with neutral balsam. Histopathological changes were observed under a light microscope.

### Cell culture and drug treatment

HFLS-RA cells were cultured in DMEM medium. The specific configuration method of DMEM medium was as follows: 10% fetal bovine serum, 100 U/mL penicillin, and 100 μg/mL streptomycin were added to the blank DMEM medium. The DMEM medium should be changed once every two to 3 days and washed with PBS once or twice before changing. HFLS-RA cells were cultured in humidified air containing 5% CO_2_ at 37 °C.

#### Cell viability assay

Cell viability was detected by MTT assay. HFLS-RA cells were inoculated into 96-well cell culture dishes and cultured overnight. Following treatment, MTT solution (0.5 mg/mL) was added to each well and incubated for 3 h at 37 °C. The supernatant was then removed, and the formazan crystals were solubilized in 100 μL DMSO per well. Absorbance was measured at 570 nm using a microplate reader (BIO-RAD 680; Thermo Fisher, MA, United States). Cell viability was expressed as a percentage relative to untreated control wells. The MTT test was repeated at least three times independently.

### RNA isolation and quantitative real-time PCR

After the cells were treated with drugs, total RNA was isolated from each group of cells according to the RNA extraction steps of the manufacturer using the RNAeasy Kit (Qiagen). CDNA was synthesized from RNA using the reverse transcription aid first strand cDNA Kit (Thermo Scientific). Using IL-1 gene primers (Forward: CGA​ATC​TCC​GAC​CAC​CAC​TA; Reverse: AGC​CTC​GTT​ATC​CCA​TGT​GT), IL-6 (Forward: AGA​CAG​CCA​CTC​ACC​TCT​TCA​G; Reverse: TTCTGCCAGTGCCTCTT -TGCTG), GAPDH (Forward: TCA​AGA​AGG​TGG​TGA​AGC​AGG; Reverse: TCA​AAG​GTG​GAG​GAG​TGG​GT). The gene expression was calculated by the 2 ^−^ΔΔ CT method, and all internal parameters were normalized to GAPDH. All experiments were repeated three independent experiments.

### LDH release assay

LDH cytotoxicity Kit (Beyotime Biotechnology, Shanghai, China) was used to evaluate cytotoxicity. In short, according to the size and growth rate of cells, an appropriate amount of cells was seeded into a 96-well cell culture plate. The HFLS-RA cells were seeded into a 96-well plate at a density of 1 × 10^5^ (100 μL per well), and cultured overnight at 37 °C and 5% CO_2_. After treatment with sanguinarine, the culture supernatant was collected by centrifugation at 400 *g* for 5 min, and 50 μL supernatant per well was transferred to a new detection plate. The LDH working solution was prepared, add 50 μL to each well, and then incubated in the dark at room temperature for 30 min. The absorbance values of each group were measured at 490 nm.

### Migration and invasion assays

First, HFLS-RA cells were cultured in serum-free medium for 12 h, and cell migration was observed. The cell suspension was added to the upper chamber of the Trans-well, and the serum-containing medium was added to the lower chamber. After 24 h of culture, the non-migrated cells on the upper surface were removed, and the cells that migrated to the lower surface were fixed with paraformaldehyde and then stained with crystal violet. The migrating cells were counted by randomly selecting the visual field under the microscope. For the invasion test, a Trans-well insert coated with Matrigel was used. HFLS-RA cells were seeded on Matrigel-coated implants. The treatment group included the sanguinarine group and the control group. The cells invading the lower surface were fixed, and then stained and counted with crystal violet.

### TUNEL assay

After drug treatment, the adherent cells seeded on the cover glass were fixed with 4% paraformaldehyde (PFA) at room temperature for 25 min, and then infiltrated with 0.3% Triton X-100 for 10 min. After washing with PBS, the cells were incubated with the TUNEL reaction mixture (prepared according to the instructions of the kit) at 37 °C in the dark for 60 min. The nuclei were re-stained with DAPI for 5 min, and then washed with PBS three times. The apoptotic cells were observed using a Zeiss 880 laser confocal microscope.

### Measurement of intracellular ROS levels

DCFH-DA probe, fluorescence microscope, and flow cytometry were used to detect the content of reactive oxygen species (ROS) in cells. In short, the cells were incubated with DCFH-DA in the incubator for 15 min at 37 °C and 5% CO_2_, and then washed twice with PBS. At the excitation/emission wavelength of 488/525 nm, the fluorescence intensity was measured with a microplate reader. Flow cytometry analysis showed that 1 × 10 ^5^ cells of each sample were stained with DCFH-DA at 37 °C in 5% CO_2_ incubator for 15 min. The cells were then filtered and immediately analyzed by flow cytometry. All experiments were repeated at least three independent repetitions.

### Measurement of mitochondrial membrane potential (Δψm)

Stain each group of cells with JC-1 dye, and then use fluorescence microscopy and flow cytometry to detect the Δψm. Fluorescence microscopy was used to detect Δψm by seeding cells into a 96-well plate with a cell density of 1 × 10^4^ cells per well. After drug treatment, the cells were incubated with JC-1 fluorescent dye (10 μg/mL) at 37 °C in the dark for 15 min, followed by washing twice with PBS. Fluorescence images were observed under a fluorescence microscope, with red fluorescence detected at excitation/emission wavelengths of 560/595 nm and green fluorescence detected at 485/535 nm. Calculate the ratio of red/green fluorescence intensity to evaluate the state of mitochondria. The flow cytometry analysis method is as follows: cells treated with drugs are stained with JC-1 for 15 min under dark conditions at 37 °C, then filtered and immediately analyzed using a flow cytometer. All experiments should be conducted in at least three independent replicates.

### Western blotting analysis

After treating each group of cells with sanguinarine, HFLS-RA cells were collected and lysed in RIPA buffer supplemented with a mixture of protease and phosphatase inhibitors. According to the manufacturer’s instructions, use the BCA protein detection kit (Thermo Scientific) to determine the protein concentration of each group. Separate equal amounts of proteins (40 μg per lane) by SDS-PAGE and transfer them onto an NC membrane. Seal the membrane with 5% BSA at room temperature for 1 h. Then, at 4 °C, the primary antibodies of Bcl-2, Bax, YAP, TAZ, and GAPDH were incubated overnight (all diluted 1:1,000; Santa Cruz Biotechnology). Retrieve the primary antibody, wash it three times with TBST, and incubate the NC membrane with HRP-conjugated secondary antibody (diluted 1:5,000; Promega) at room temperature for 1 h. Visualize protein bands using enhanced chemiluminescence (ECL) reagents. Quantify band intensity using ImageJ software (NIH). All experiments were conducted in at least three independent replicates.

### Immunofluorescence analysis

Establish a control group, and treat cells with different concentrations of sanguinarine in the experimental group. The treated cells were fixed with 4% PFA for 10 min, blocked with 3% BSA at room temperature for 1 h, and incubated overnight with primary antibody at 4 °C. After three washes with PBS, the cells were incubated with the corresponding secondary antibody at 37 °C. Subsequently, the nuclei were labeled with DAPI, and fluorescence images of the control and experimental groups were collected using a Zeiss laser confocal microscope.

### Immunohistochemical staining

Decalcified hind leg slices and synovial tissue slices of rats were deparaffinized and boiled in a recovery buffer at 100 °C for 20 min for antigen recovery. Endogenous peroxidase activity was blocked with 3% hydrogen peroxide, and then non-specific binding was blocked with normal goat serum. Slices were incubated overnight with the corresponding primary antibody at 4 °C, then washed three times with PBS and incubated with horseradish peroxidase (HRP) conjugated secondary antibody at 37 °C for 1 h. After three washes with PBS, the slices were visualized using DAB chromogenic solution, counterstained with hematoxylin, dehydrated, removed, and fixed with neutral resin. Observe and photograph the staining results under an optical microscope.

### Quantitative analysis of immunofluorescence, immunohistochemical staining and Western blotting

Briefly, ImageJ software was applied to quantify all staining and blot signals. Immunofluorescence levels were assessed via mean fluorescence intensity (MFI), and immunohistochemical positive staining was quantified based on average optical density (AOD). Western blot band gray values were normalized to housekeeping proteins to correct unequal protein loading.

### Statistical analysis

All data are presented as mean ± SEM. Statistical analyses were performed using GraphPad Prism 8.0 software. Comparisons between two groups were made using a two-tailed Student’s t-test. For multiple group comparisons, one-way analysis of variance (ANOVA) was used, followed by Tukey’s *post hoc* test for pairwise comparisons. *P* values < 0.05 were considered statistically significant. All experiments were independently repeated at least three times.

## Results

### Sanguinarine suppresses inflammation and bone erosion in adjuvant-induced arthritis

To evaluate the therapeutic efficacy of sanguinarine against RA, we applied an adjuvant-induced arthritis (AIA) model in SD rats. The study consisted of six experimental groups: healthy controls, AIA-induced arthritic rats (model group), a positive-control group treated with methotrexate (MTX; 7.5 mg/kg/week, p.o.), and three treatment groups receiving sanguinarine at doses of 2.5, 5, and 10 mg/kg. Therapeutic outcomes were measured by four key parameters: clinical inflammation scores, paw swelling, radiographic evidence of bone destruction, and elevated serum cytokine levels (Figure 1A). The AIA model was effectively induced, with disease onset apparent by day 9 and reaching a 100% incidence of joint inflammation by day 15. The results demonstrated that the sanguinarine groups exhibited a higher weight, reaching an average of 220 g *versus* 198 g in the model group (Figure 1B). Moreover, sanguinarine treatment exhibited a dose-dependent reduction in arthritis foot scores, as shown in Figure 1C. The measurement of foot swelling volume and radiographic analyses revealed that sanguinarine conferred marked protection against AIA-induced joint swelling and bone erosion. Specifically, sanguinarine treatment groups at doses of 2.5, 5, and 10 mg/kg exhibited a distinctly smaller increase in paw swelling (1.76, 0.84 and 0.62, respectively) compared with the untreated AIA model group (2.46) (Figures 1D,E). Moreover, the therapeutic efficacy of the 5 mg/kg sanguinarine group was equivalent to that of methotrexate. Thus, this concentration was selected for all subsequent biochemical and functional analyses. In addition, Safranin O and H&E staining of joint sections further demonstrated preserved cartilage architecture and reduced inflammatory infiltration in treated groups, confirming the bone-protective effect of sanguinarine (Figure 1F).

**FIGURE 1:**
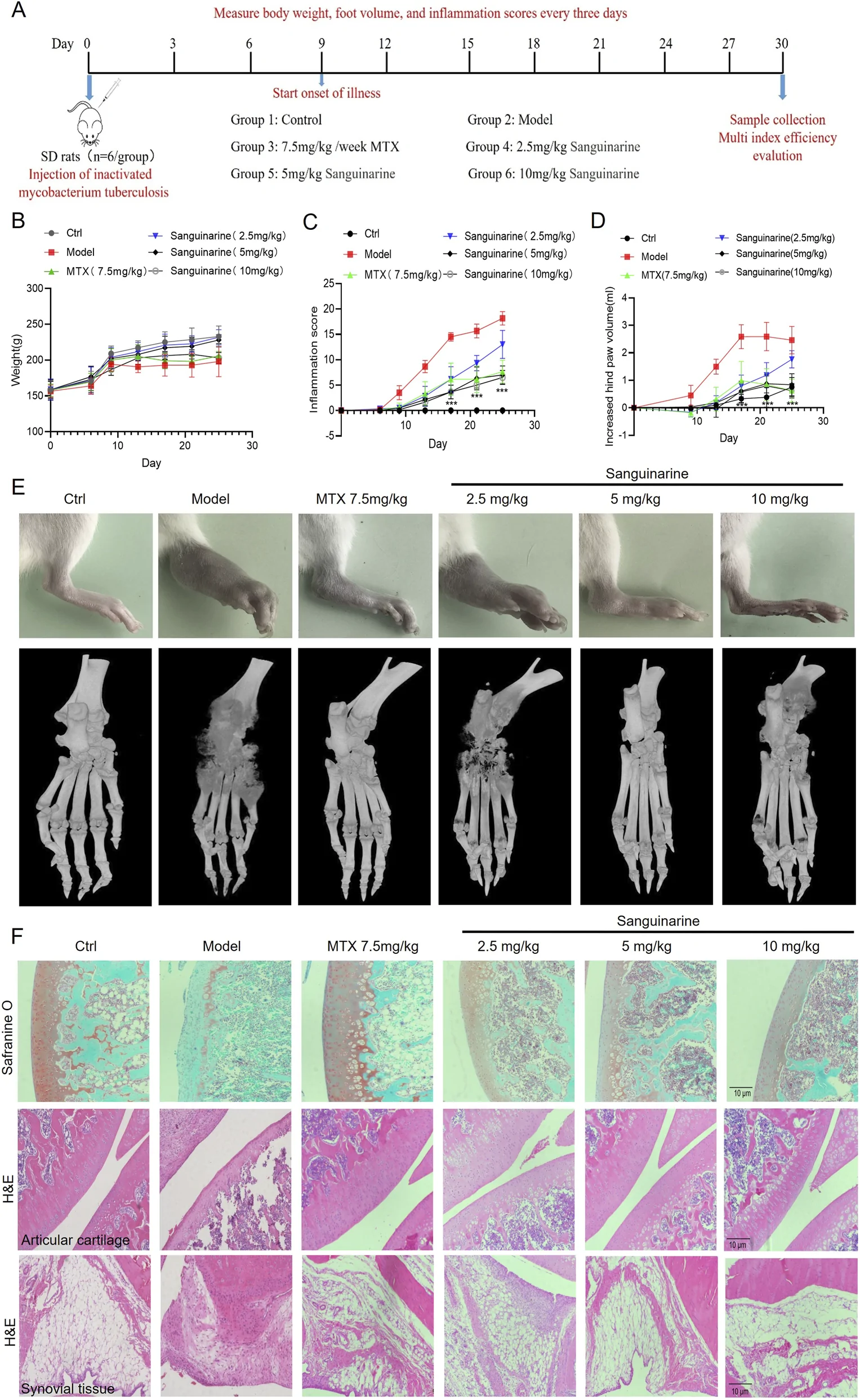
Sanguinarine has anti-inflammatory and anti-bone destruction effects on AIA. **(A)** Schematic Diagram of Animal Modeling and Drug Delivery Administration Process. **(B)** Body weight measurements over the experimental timeline. **(C)** The arthritis score of rats was assessed at the indicated time points. **(D)** Hind-paw volume determined by plethysmometry. **(E)** The destruction of the joint bones of rats was measured by X-Ray. **(F)** Histological evaluation of right hind-limb joint sections stained with H&E and Safranin-O/Fast Green. n = 6 for each group. Quantitative measurements were completed by investigators blinded to the experimental group assignments *p < 0.05, **p < 0.01, ***p < 0.001 vs. model group.

### Sanguinarine attenuates adjuvant-induced arthritis by suppressing inflammatory responses and synovial apoptosis

In the pathogenesis of rheumatoid arthritis, innate immune cells infiltrate the synovium and secrete a series of key pro-inflammatory cytokines, including tumor necrosis factor -α (TNF-α), interleukin-1 (IL-1), and interleukin-6 (IL-6). These key mediators can impair the activity and physiological functions of chondrocytes, leading to pathological synovial hyperplasia and triggering self-sustaining cascade reactions, exacerbating joint inflammation and irreversible structural damage. Accordingly, targeted modulation of the aberrant inflammatory response has emerged as the cornerstone of therapeutic strategies for arthritis. As shown in Figure 2A, the spleen index (organ wet weight (mg)/animal body weight (g)×100%) was significantly higher in the model group compared to the controls, whereas the spleen index in the sanguinarine group tended to normalize, indicating that sanguinarine treatment effectively reduces splenomegaly and injury. Additionally, the thymus and liver indices in the treatment groups were comparable to those in the model group and showed no statistically significant differences, indicating that sanguinarine has no obvious toxic side effects (Figures 2B,C). ELISA-based quantification of serum inflammatory factors revealed that the levels of IL-1β, PGE2, IL-6, and TNF-α were drastically increased in the model group relative to the normal control group. Critically, sanguinarine treatment significantly blunted the high levels of TNF-α, IL-1β, PGE2, and IL-6 compared with the model group (Figures 2D–G). These findings indicate that sanguinarine has significant anti-inflammatory properties, and its efficacy is enhanced in a dose-dependent manner. Finally, we performed TUNEL staining and caspase-3 activity assays to assess apoptosis in joint synovial cells. The results show that sanguinarine treatment increased the number of apoptotic cells in the AIA rat model (Figures 2H,I). In summary, sanguinarine suppresses the inflammatory cascade and synovial cell apoptosis, thereby ameliorating arthritic pathology.

**FIGURE 2:**
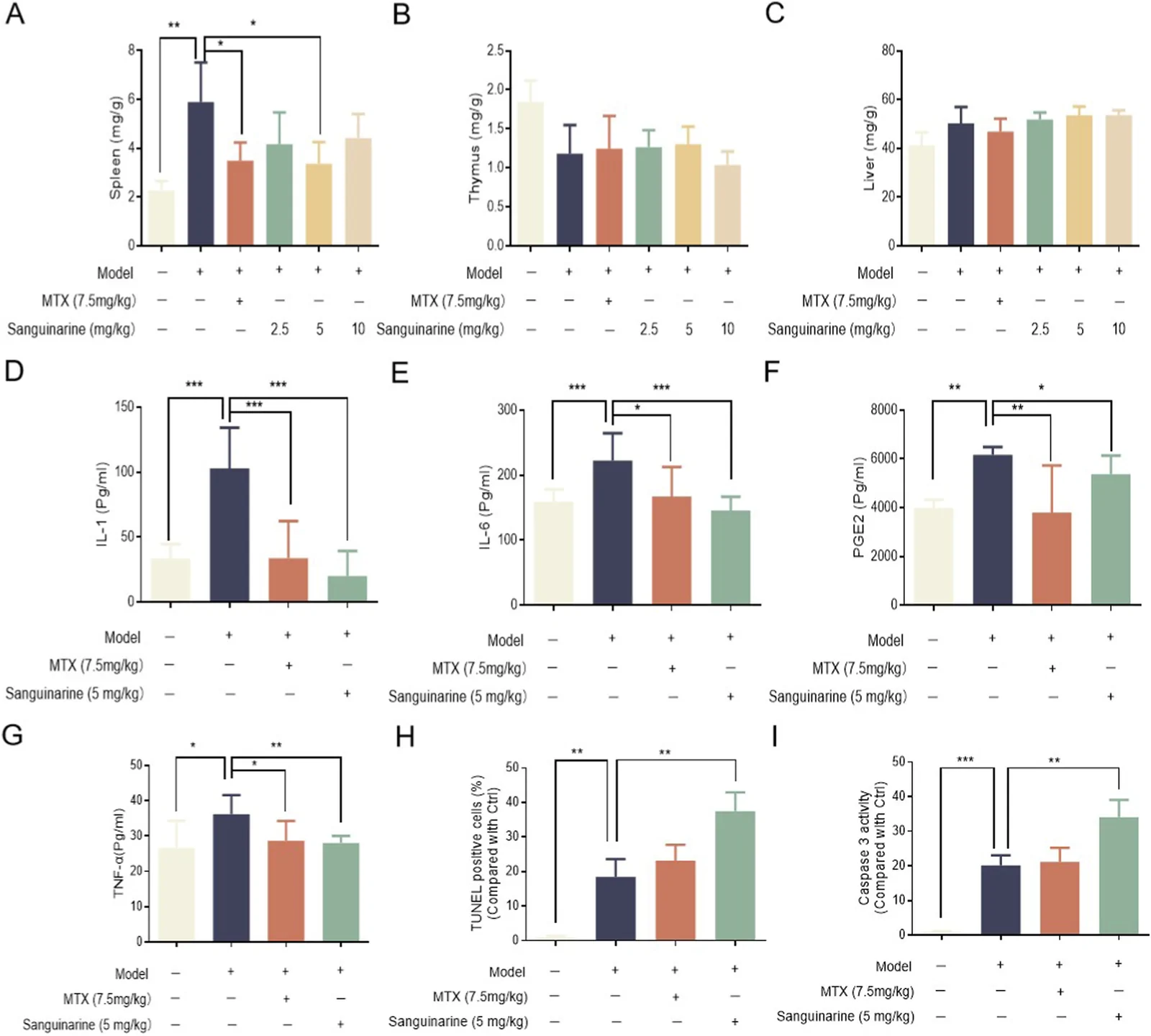
Sanguinarine Attenuates Adjuvant-Induced Arthritis by Suppressing Inflammatory Responses and Synovial Cell Apoptosis. **(A–C)** Organ index of spleen, thymus, and liver in each group. **(D–G)** Serum levels of IL-1β, IL-6, PGE2, and TNF-α were quantified by ELISA. **(H)** The TUNEL-positive cells of rats were detected by TUNEL staining. **(I)** Caspase-3 protein levels in rat joint tissues using commercially available assay kits. n = 6 for each group. Quantitative measurements were completed by investigators blinded to the experimental group assignments *p < 0.05, **p < 0.01, ***p < 0.001 vs. model group.

### Sanguinarine triggers cell death and mitigates pro-inflammatory activation in HFLS-RA cells

To investigate the mechanism of action of sanguinarine against arthritis, we used HFLS-RA cells. To evaluate the cytotoxic effects of sanguinarine, we assessed cell viability and lactate dehydrogenase (LDH) release in HFLS-RA cells. Cells were stimulated with TNF-α for 3 h before treatment with increasing concentrations of sanguinarine for 24 h, and viability was measured by MTT assay. Compared to control conditions, sanguinarine at concentrations ranging from 2.5 μM to 5 μM induced pronounced cell death in HFLS-RA cells (Figure 3A). For HFLS-RA cells co-treated with TNF-α and sanguinarine, the cell viability at each sanguinarine concentration showed no statistically significant difference (*ns*) relative to the corresponding single sanguinarine groups at matching doses. Furthermore, a dose-dependent elevation in LDH release was observed in cells treated with sanguinarine at concentrations of 2.5 and 5 μM when compared to the control group (Figure 3B). Given their capacity to invade adjacent articular cartilage, fibroblast-like synoviocytes (FLS) in RA exhibit neoplastic-like properties, we further examined the effect of sanguinarine on HFLS-RA cell migration and invasion. Sanguinarine at concentrations of 1.25, 2.5, and 5 μM inhibited the migration and invasion of HFLS-RA cells (Figures 3C–E). To further validate the anti-inflammatory activity of sanguinarine, we quantified the mRNA expression of *IL-1* and *IL-6* following 24 h treatment. Based on the above cell viability and cytotoxicity data, only 2.5 μM and 5 μM sanguinarine were selected for cytokine detection, as the 1.25 μM group showed negligible cytotoxicity toward HFLS-RA cells. Sanguinarine markedly downregulated the transcript abundance of these pro-inflammatory cytokines in HFLS-RA cells (Figures 3F,G).

**FIGURE 3:**
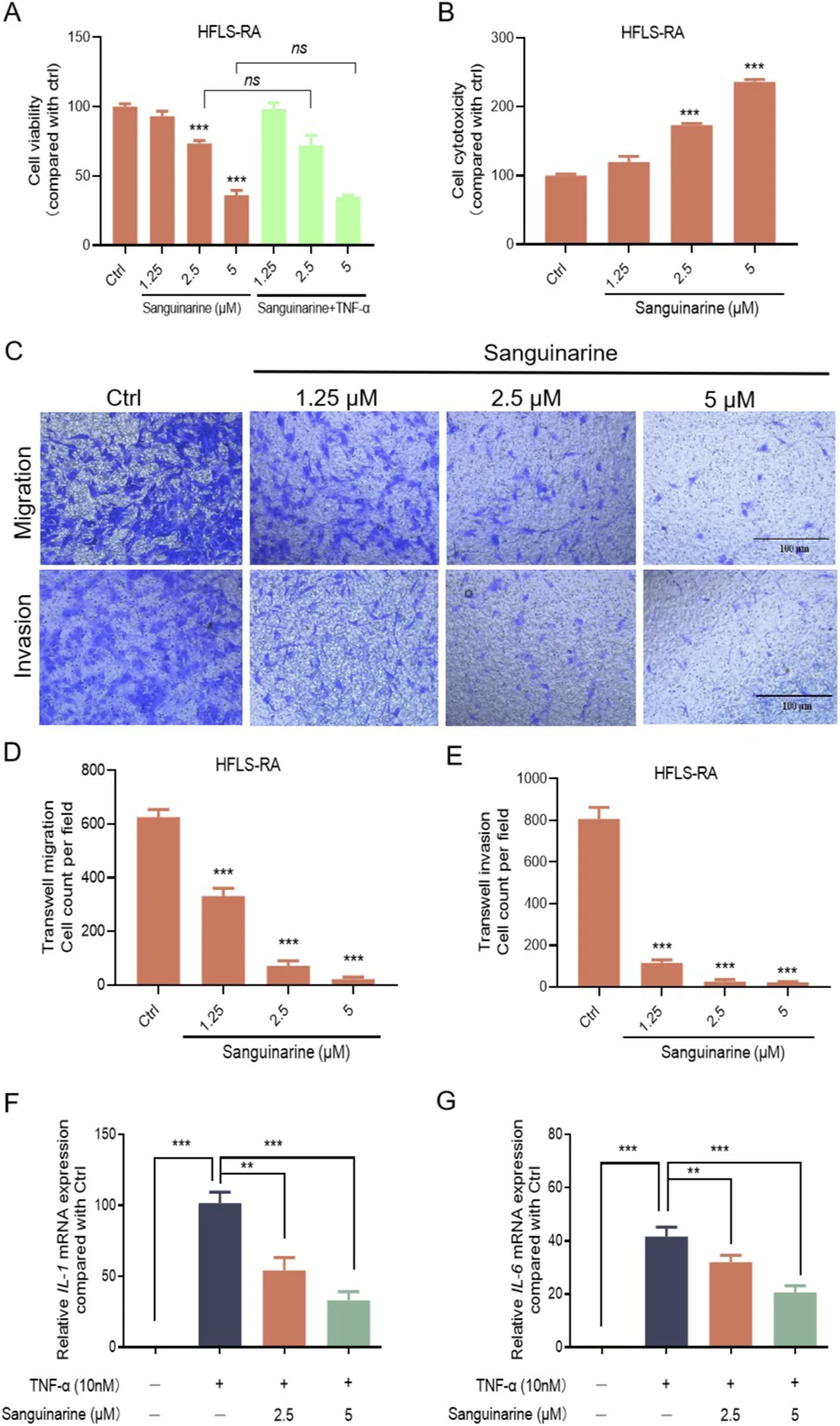
Sanguinarine triggers cell death and mitigates pro-inflammatory activation in HFLS-RA cells. **(A)** Cell viability of HFLS-RA cells in 24 h by MTT assay. **(B)** The effect of sanguinarine on LDH release in HFLS-RA cells. **(C–E)** Migration and invasion assays were performed after different doses of sanguinarine treatment. **(F, G)** mRNA levels of cellular inflammatory factors IL-1 and IL-6 were measured by qRT-PCR. n = 3 for each group. Data were analyzed as mean ± SEM for triplicate experiments (*, *p* < 0.05 compared with control group; *P < 0.05, **P < 0.01, ***P < 0.001, “*ns*” stands for “not statistically significant”).

### Treatment with sanguinarine triggered cell apoptosis in the HFLS-RA cell line

The pro-apoptotic effect of sanguinarine on HFLS-RA cells was further examined using a range of assays. After 24 h of treatment with increasing doses of sanguinarine, cells were harvested, and apoptosis was quantified by TUNEL staining, caspase-3 activity measurement, Western blotting, and flow cytometry. According to the results of TUNEL staining, as shown in Figures 4A,B, sanguinarine caused remarkable cell apoptosis. Consistent with these findings, flow cytometry analysis revealed a significant increase in apoptotic cells following sanguinarine treatment (Figures 4C,D). To further verify the pro-apoptotic effect of sanguinarine, we measured caspase-3 activity, a key executioner of apoptosis. Treatment with sanguinarine increased caspase-3 activity in a dose-dependent manner (Figure 4E). Consistent with this, sanguinarine treatment significantly upregulated the Bax/Bcl-2 expression ratio, further indicating activation of the mitochondrial apoptotic pathway (Figures 4F,G). To investigate the mechanism underlying sanguinarine-induced apoptosis in HFLS-RA cells, we examined its effects on mitochondrial function, given the established role of mitochondrial dysfunction and reactive oxygen species (ROS) in autoimmune diseases such as rheumatoid arthritis (RA). We first assessed mitochondrial membrane potential (ΔΨm) using the JC-1 assay. Treatment with increasing concentrations of sanguinarine (1.25, 2.5, and 5 μM) for 24 h resulted in a dose-dependent decrease in the red/green fluorescence ratio, indicating ΔΨm disruption (Figures 4H,I). We next evaluated whether this mitochondrial impairment was associated with ROS production. Using the DCFH-DA probe, we found that sanguinarine treatment for 4 h induced a dose-dependent increase in intracellular ROS levels (Figures 4J,K). Moreover, treatment with 5 μM sanguinarine led to a time-dependent accumulation of ROS over 4 h (Figures 4L,M).

**FIGURE 4:**
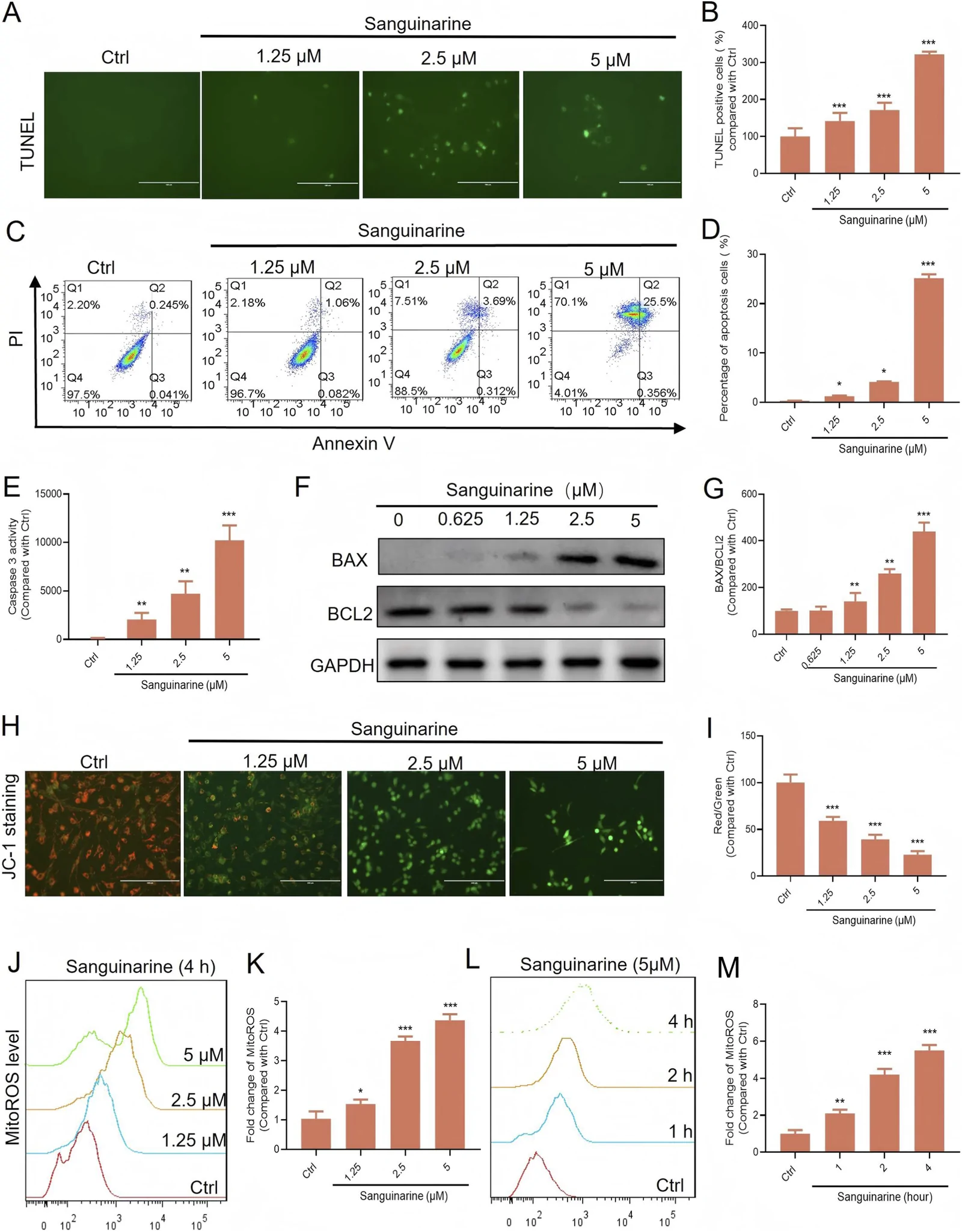
Sanguinarine treatment induced cell apoptosis in the HFLS-RA cell line. **(A,B)** Cell apoptosis was detected by TUNEL staining with sanguinarine treatment at different doses, bar = 100 μm. **(C,D)** Cell apoptosis was detected by flow cytometry with sanguinarine treatment at different doses. **(E)** Caspase 3 activity was detected using commercially available assay kits. **(F,G)** Dose-dependent effects of sanguinarine on the Bax/Bcl-2 ratio in HFLS-RA cells. **(H,I)** Mitochondrial membrane potential was detected by the JC-1 assay with sanguinarine treatment at different doses. **(J,K)** ROS was detected by flow cytometry with sanguinarine treatment at different doses. **(L,M)** ROS was detected by flow cytometry with sanguinarine treatment at different times. n = 3 for each group. Data analyzed as mean ± SEM for triplicate experiments (*p < 0.05 compared with control group; *P < 0.05, **P < 0.01, ***P < 0.001).

### Sanguinarine mitigates rheumatoid arthritis via modulation of the Hippo-YAP/TAZ signaling cascade

The transcriptional coactivators YAP and TAZ play key roles in regulating the proliferation, migration, and survival of chondrocytes and FLSs. Their activity, controlled by the Hippo signaling pathway, is critical for maintaining joint homeostasis, and disruption of this balance can result in cartilage pathology (Caire et al., 2021; Li et al., 2024; Lu et al., 2024). However, the involvement of the Hippo-YAP axis in arthritis pathogenesis remains poorly understood. We therefore sought to determine whether sanguinarine exerts its anti-arthritic effects in HFLS-RA cells by modulating YAP/TAZ transcriptional activity. Treatment with sanguinarine increased the phosphorylation of MST1/2, LATS1/2, YAP, and TAZ in both a time- and dose-dependent manner, indicating activation of the Hippo kinase cascade (Figures 5A–E). Consistent with this, immunofluorescence analysis revealed that sanguinarine treatment reduced nuclear localization of YAP and TAZ, promoting their cytoplasmic retention (Figures 5F,G). These results suggest that sanguinarine suppresses YAP/TAZ transcriptional activity by promoting their nuclear exclusion via Hippo pathway activation.

**FIGURE 5:**
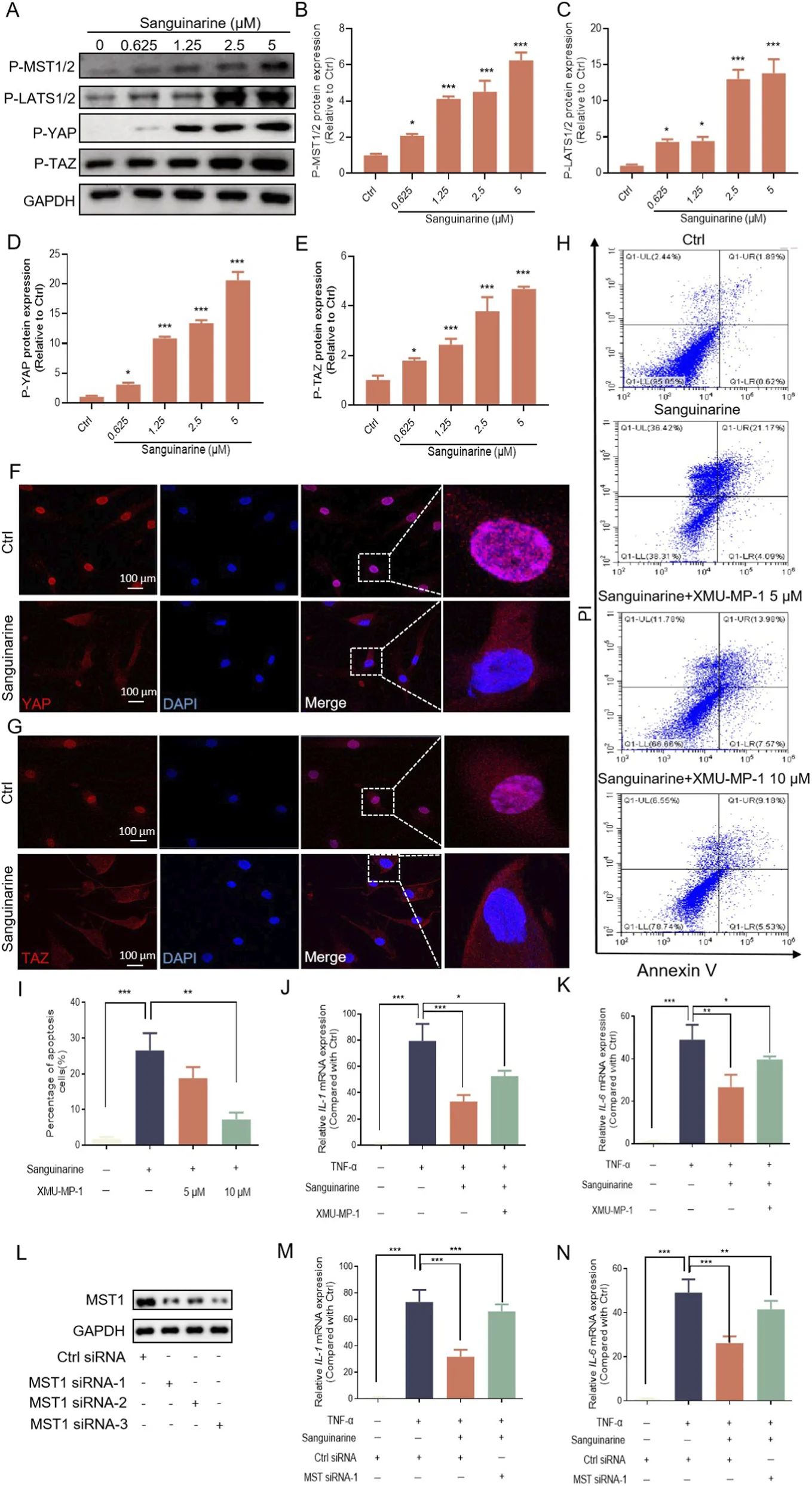
Sanguinarine ameliorates rheumatoid arthritis by regulating the Hippo-YAP/TAZ pathway. **(A–E)** Western blot was used to detect the expression of Hippo-YAP/TAZ-related proteins in HFLS-RA cells treated with different concentrations of sanguinarine. **(F)** Immunofluorescence analysis of YAP nuclear localization with sanguinarine treatment in HFLS-RA. Red fluorescence represented images of YAP, and blue fluorescence represented cell nuclei. **(G)** Immunofluorescence analysis of TAZ nuclear localization with sanguinarine treatment in HFLS-RA. Red fluorescence represented images of TAZ, and blue fluorescence represented cell nuclei. **(H,I)** Apoptosis of HFLS-RA cells treated with sanguinarine alone or in combination with XMU-MP-1 was assessed by flow cytometry. **(J,K)** mRNA levels of *IL-1* and *IL-6* in HFLS-RA cells treated with sanguinarine alone or in combination with XMU-MP-1 were assessed by qPCR. **(L)** Western blotting was performed to assess MST1 silencing efficiency. **(M,N)** mRNA levels of *IL-1* and *IL-6* in HFLS-RA cells treated with sanguinarine alone or co-transfected with MST1 siRNA were assessed by qPCR. n = 3 for each group. Data were analyzed as mean ± SEM for triplicate experiments (*P < 0.05, **P < 0.01, ***P < 0.001).

We next tested whether MST1/2, the Hippo pathway upstream kinase, is essential for the anti-arthritic effects of sanguinarine. Inhibition of MST1/2 with XMU-MP-1 (5 μM) effectively blocked the apoptotic response to sanguinarine treatment in HFLS-RA cells, as measured by flow cytometry (Figures 5H,I). Consistent with this, XMU-MP-1 treatment also reversed the inhibitory effect of sanguinarine on *IL-1* and *IL-6* expression (Figures 5J,K). More importantly, transfection with MST1 siRNA reversed the anti-inflammatory effects of sanguinarine, as evidenced by the abrogation of sanguinarine-mediated suppression of *IL-1* and *IL-6* expression (Figures 5L–N). These results establish MST1/2 as a key mediator of sanguinarine therapeutic effects in HFLS-RA cells.

### Treatment with an ROS scavenger reversed sanguinarine’s protective phenotype as well as its capacity to activate Hippo signaling in HFLS-RA cell line

To further verify the regulatory mechanism of sanguinarine, we performed rescue experiments using the ROS inhibitor N-acetyl-L-cysteine (NAC). HFLS-RA cells were pretreated with 5 mM NAC for 1 h, followed by co-treatment with sanguinarine. As shown in Figures 6A,B, NAC supplementation effectively reversed the intracellular ROS accumulation induced by sanguinarine. JC-1 staining combined with flow cytometry was further applied to evaluate alterations in mitochondrial membrane potential. Consistent with the ROS scavenging effect of NAC, NAC pretreatment significantly rescued sanguinarine triggered mitochondrial membrane potential loss (Figures 6C–E). Furthermore, flow cytometric apoptosis detection and caspase 3 activity assays demonstrated that NAC administration largely abrogated sanguinarine mediated apoptosis in HFLS RA cells (Figures 6F–H). Importantly, Treatment with NAC also reversed the activating effect of sanguinarine on Hippo signaling in HFLS-RA cells (Figures 6I,J). Collectively, these findings indicate that ROS elevation serves as an essential upstream driver of sanguinarine induced apoptosis and Hippo pathway activation in HFLS-RA cells.

**FIGURE 6:**
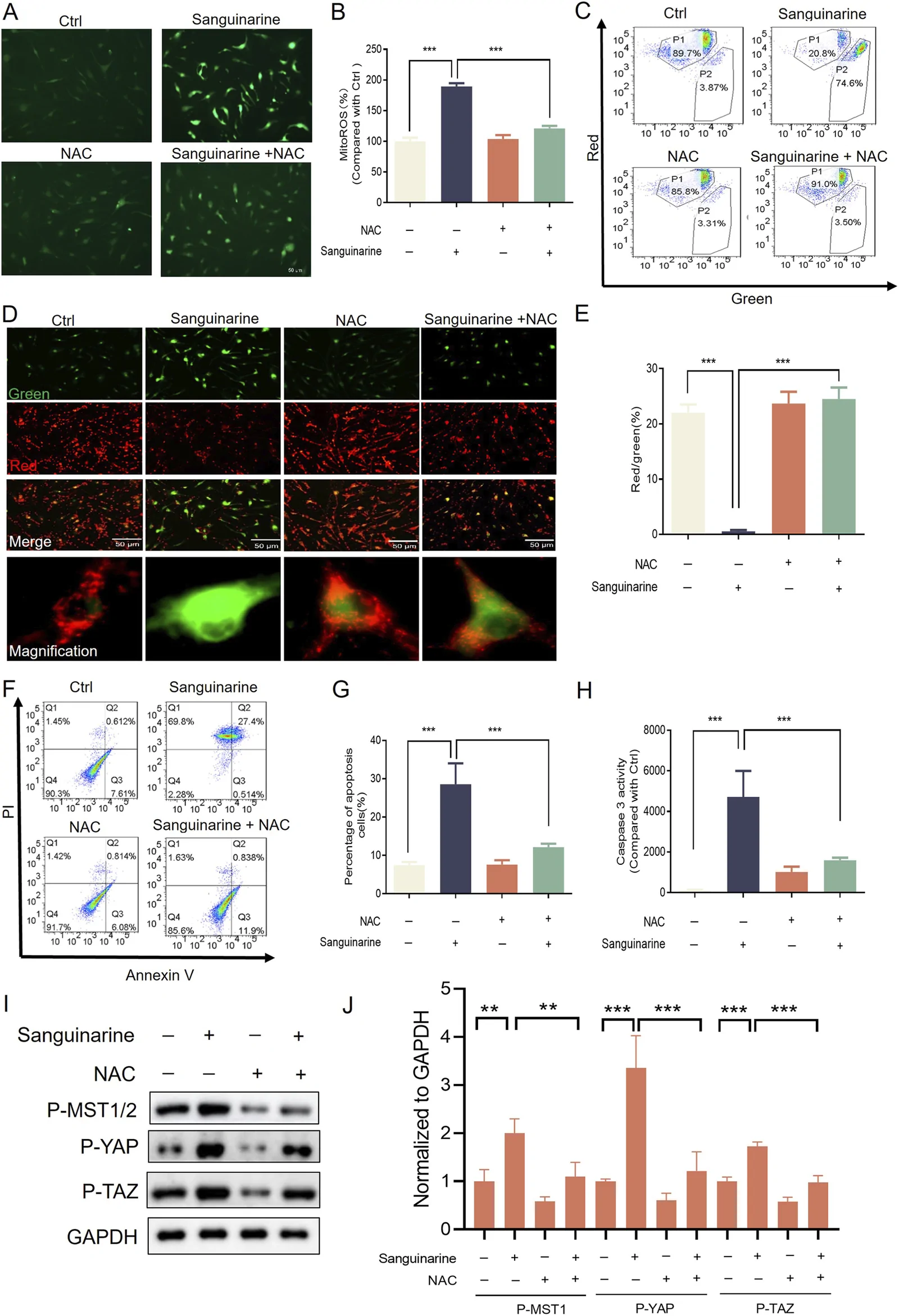
Treatment with an ROS scavenger reversed sanguinarine’s protective phenotype as well as its capacity to activate Hippo signaling in the HFLS-RA cell line. **(A,B)** Representative images and quantification of MitoROS levels in HFLS-RA cells treated with sanguinarine alone or combined with NAC. **(C)** Flow cytometry analysis of mitochondrial membrane potential in HFLS-RA cells treated with sanguinarine alone or combined with NAC. **(D,E)** Quantitative analysis of JC-1 staining in HFLS-RA cells treated with sanguinarine alone or combined with NAC. **(F,G)** Flow cytometry was used to quantify apoptotic HFLS-RA cells following treatment with sanguinarine alone or in combination with NAC. **(H)** Caspase 3 activity was detected in HFLS-RA cell treated with sanguinarine alone or combined with NAC. **(I,J)** Western blot analysis and quantification of P-MST1/2, P-YAP, P-TAZ protein levels in HFLS-RA cell treated with sanguinarine alone or combined with NAC. n = 3 for each group. Data analyzed as mean ± SEM for triplicate experiments (*P < 0.05, **P < 0.01, ***P < 0.001).

### Hippo-YAP/TAZ signaling pathway was involved in the anti-arthritic effects of sanguinarine *in vivo*

To validate the involvement of the Hippo signaling pathway in the anti-arthritic effects of sanguinarine *in vivo*, we performed immunohistochemical analysis in a rat model of RA. In vehicle-treated RA mice, phosphorylation of MST1/2, LATS1/2, YAP, and TAZ was reduced compared to healthy controls. Notably, sanguinarine treatment reversed this phenotype, significantly increasing the phosphorylation levels of these Hippo pathway components (Figures 7A–E). These results demonstrate *in vivo* that sanguinarine ameliorates RA pathology by modulating the Hippo-YAP/TAZ signaling pathway.

**FIGURE 7:**
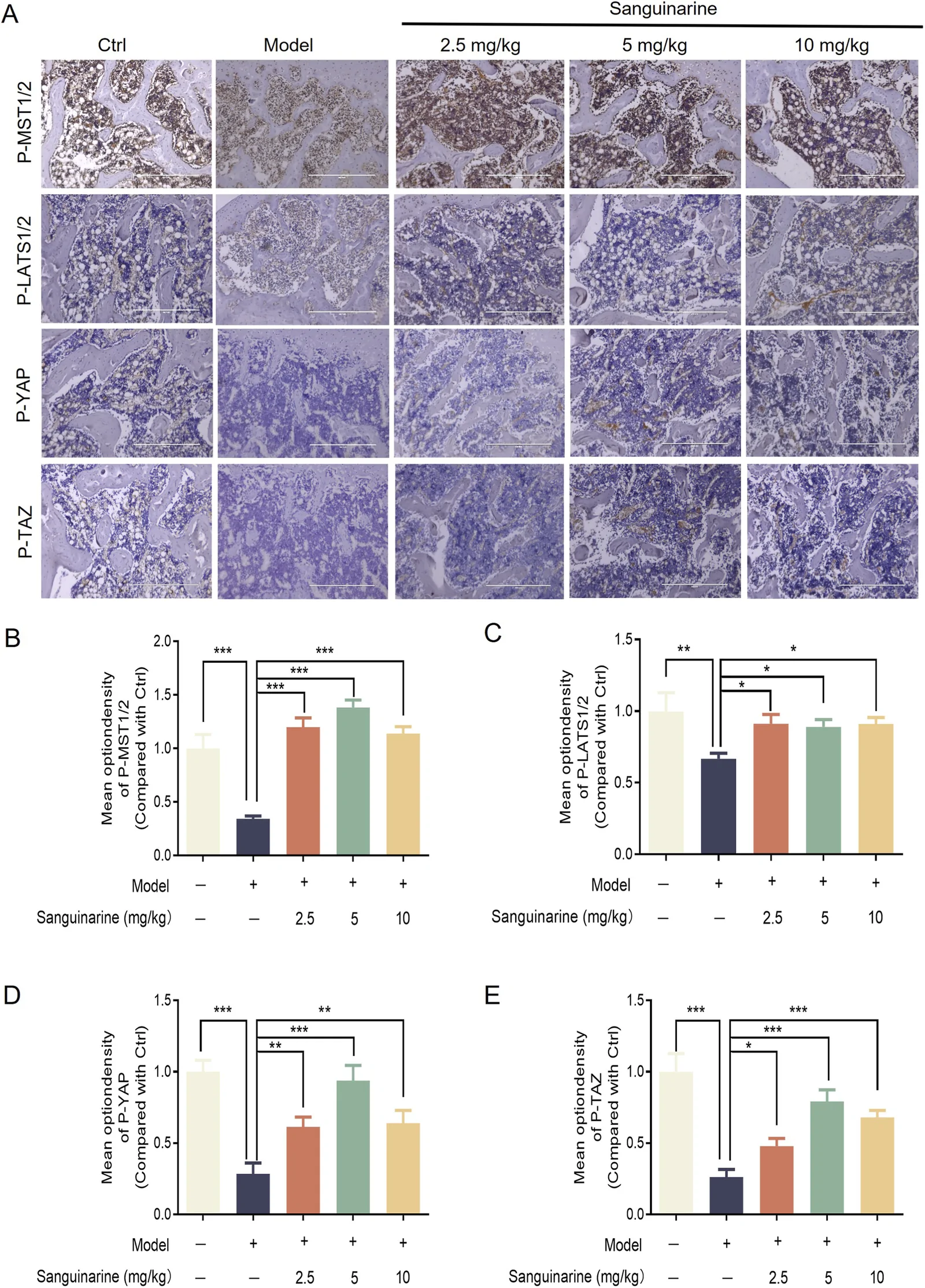
Hippo-YAP/TAZ signaling pathway was involved in the anti-arthritic effects of sanguinarine *in vivo*. **(A)** Immunohistochemical (IHC) staining was performed to evaluate the expression levels of P-MST1/2, P-LAST1/2, P-YAP, and P-TAZ in each group. **(B–E)** Quantification of P-MST1/2, P-LAST1/2, P-YAP, and P-TAZ in each group. n = 6 for each group (*P < 0.05, **P < 0.01, ***P < 0.001).

## Discussion

RA is a chronic autoimmune disease that can occur at any age. The current clinical management mainly relies on drugs that alleviate inflammation and symptoms, but effective disease improvement therapies are still limited. Traditional Chinese medicine (TCM) has a long history in the treatment of RA, providing symptom relief through multi-target mechanisms (Li et al., 2023; Liang et al., 2023; Wang et al., 2021). Sanguinarine, a benzophenanthridine-type alkaloid obtained from TCM, displays multiple biological properties, including anti-inflammatory, antioxidant, antitumor, and antimicrobial effects. Nevertheless, whether sanguinarine possesses anti-arthritic effects and the molecular basis underlying such actions remains largely unknown. In the present work, we revealed that sanguinarine promotes apoptosis and suppresses inflammation in both the AIA rat model and HFLS-RA cells. Moreover, we uncovered that sanguinarine alleviates rheumatoid arthritis through regulation of the Hippo-YAP/TAZ signaling pathway.

Collagen-induced arthritis (CIA) and complete CFA-induced arthritis (AIA) are two well-established animal models commonly utilized in RA research. These models differ considerably in their induction protocols, pathological features, and experimental applications. The CIA model is widely regarded for its capacity to closely replicate the chronic, progressive joint erosion characteristic of human RA. In contrast, the AIA model, elicited by a single CFA injection, is distinguished by its technical simplicity and rapid inflammatory onset, making it particularly suitable for preliminary screening of anti-inflammatory agents and for mechanistic studies of acute arthritis (Ali et al., 2026; Kollias et al., 2011; Razaq et al., 2026; Yan et al., 2023). Based on these practical advantages, the present study employed the AIA rat model to conduct an initial evaluation of the anti-inflammatory efficacy and potential mechanisms of sanguinarine. In the present study, we investigated for the first time the anti-arthritic effects of sanguinarine using the CFA-induced AIA rat model. Sanguinarine treatment significantly reduced arthritis scores, attenuated inflammatory responses, and decreased spleen organ indices in arthritic rats. These effects were associated with reduced plasma levels of the pro-inflammatory cytokines TNF-α, IL-1, PGE2, and IL-6.

Oxidative stress, resulting from either excessive free radical production or impaired antioxidant defense, has been implicated in the pathophysiology of various diseases, including RA (Guo et al., 2025; Wu et al., 2022). Altered oxidative status has been documented in the serum of RA patients and in tissues of experimental arthritis models. Concurrently, pro-inflammatory cytokines such as TNF-α, IL-1β, and IL-6 play pivotal roles in driving synovial inflammation (Arnold et al., 2022; Biesemann et al., 2023; Qin et al., 2022). Together, these observations establish ROS and inflammatory cytokines as key contributors to RA pathogenesis. Mitochondria serve as central hubs for apoptotic signaling, releasing pro-apoptotic proteins upon permeabilization. ROS produced within mitochondria are critical triggers of apoptotic cell death, acting in part by directly inducing mitochondrial permeability transition and subsequent loss of mitochondrial membrane potential (Δψm) (Vringer and Tait, 2023). Consistent with this paradigm, we observed that sanguinarine disrupts Δψm and induces ROS production in HFLS-RA cells. These findings recapitulate previous reports demonstrating that sanguinarine exerts anti-cancer effects via ROS-mediated mitochondrial dysfunction, and extend this mechanism to the context of rheumatoid arthritis.

Recent studies have established the Hippo signaling pathway as a key player in RA pathogenesis through multiple mechanisms. For example, YAP promotes synovial angiogenesis via Ezrin-mediated nuclear translocation and PI3K/Akt signaling (Chen et al., 2021), drives inflammatory cytokine and chemokine production, and regulates FLS autophagy and migration (Taniguchi et al., 2015). Moreover, previous studies have demonstrated that the core Hippo kinase MST1 can be activated by oxidative stress in RA, which induces mitochondrial dysfunction and cellular apoptosis via suppressing the AMPK-Sirt1 signaling axis (Wang et al., 2020). Consistent with these findings, our results further confirmed that NAC-mediated ROS depletion abrogated sanguinarine induced Hippo pathway activation in HFLS-RA cells. Collectively, these findings implicate the Hippo-YAP/TAZ axis as a central regulator of RA pathology and ROS elevation acts as an indispensable upstream regulator.

In the present study, we demonstrate that sanguinarine targets the Hippo-YAP/TAZ pathway by modulating the pro-apoptotic and anti-inflammatory effects, offering a mechanistic basis for its anti-arthritic effects.

## Data Availability

The raw data supporting the conclusions of this article will be made available by the authors, without undue reservation.
